# The methanol and ethanol solvates of 4-glutarato-*N*,*N*-diiso­propyl­tryptamine

**DOI:** 10.1107/S2056989022009094

**Published:** 2022-09-22

**Authors:** Marilyn Naeem, Barbara E. Bauer, Andrew R. Chadeayne, James A. Golen, David R. Manke

**Affiliations:** a University of Massachusetts Dartmouth, 285 Old Westport Road, North Dartmouth, MA 02747, USA; b CaaMTech, Inc., 58 East Sunset Way, Suite 209, Issaquah, WA 98027, USA; Venezuelan Institute of Scientific Research, Venezuela

**Keywords:** crystal structure, tryptamines, indoles, zwitterions, hydrogen bonds

## Abstract

The solid-state structures of two solvated forms of 4-glutarato-*N*,*N*-diiso­propyl­tryptamine were determined by single-crystal X-ray diffraction, namely, 5-[(3-{2-[bis(propan-2-yl)azaniumyl]ethyl}-1*H*-indol-4-yl)oxy]-5-oxopentanoate meth­anol monosolvate, C_21_H_30_N_2_O_4_·CH_3_OH, and the analogous ethanol monosolvate, C_21_H_30_N_2_O_4_·C_2_H_6_O. In both compounds, the 4-glutarato-*N*,*N*-di­iso­­pro­pyl­tryptamine exists as a zwitterion with a protonated tertiary ammonium and a deprotonated glutarato carboxyl­ate. The tryptamine zwitterions and alcohol solvates in both structures combine to produce near identical hydrogen-bonding networks, with N—H⋯O and O—H⋯O hydrogen bonds joining the mol­ecules together in two-dimensional networks parallel to the (100) plane.

## Abstract 2

The structures of two solvates of the zwitterionic prodrug of the psychedelic 4-HO-DiPT are reported.

## 1. Chemical context

Psychedelic compounds continue to be a major research focus for treating conditions including depression, post-traumatic stress disorder (PTSD), Alzheimer’s disease, and chronic pain (Carhart-Harris & Goodwin, 2017 ▸; Krediet *et al.*, 2020 ▸; Vann Jones & O’Kelly, 2020 ▸; Ramaekers *et al.*, 2021 ▸). Tryptamine compounds with chemical structures resembling that of the active product of magic mushrooms, psilocin (4-hy­droxy-*N*,*N*-di­methyl­tryptamine; 4-HO-DMT), are of particular inter­est. This is due not just to their structural similarities to the neurotransmitter serotonin (5-hy­droxy­tryptamine; 5-HT), but because many have desirable drug characteristics including oral availability, lowered susceptibility to mono­amine oxidase (MAO) degradation, and short duration of action (Kuypers *et al.*, 2019 ▸). The synthesis of prodrugs that undergo hydrolysis to produce 4-hy­droxy derivatives of di­alkyl­tryptamines are of increasing inter­est (Klein *et al.*, 2021 ▸; Chadeayne *et al.*, 2019*a* ▸; Chadeayne, Pham, Reid *et al.*, 2020 ▸; Naeem *et al.*, 2022 ▸).

4-Hy­droxy-*N*,*N*-diiso­propyl­tryptamine (4-HO-DiPT) is one example of a psilocin analog, first synthesized in 1977, in which both methyl groups on the ethyl­amino moiety of psilocin are replaced with isopropyl groups (Repke *et al.*, 1977 ▸). In early 2022, 4-HO-DiPT along with four other psychedelics were part of a proposal issued by the US Drug Enforcement Administration (DEA), requesting comments on reclassifying these compounds to Schedule I of the Controlled Substance Act. Due to a strong public response, the DEA withdrew the proposal before the hearing, which was scheduled for August (US DEA, January 14 & July 6, 2022*a* ▸,*b* ▸).

4-HO-DiPT is a serotonin-2A (5-HT_2A_) receptor agonist that, like psilocin, produces a head-twitch response (HTR) in mice, indicating its competence in producing psychedelic effects (Halberstadt *et al.*, 2020 ▸). 4-HO-DiPT also inter­acts with the serotonin transporter (SERT) with IC_50_ values in the low micromolar range, similar to 3,4-methyl­ene­dioxy­methamphetamine (MDMA) (Rickli *et al.*, 2016 ▸). 4-HO-DiPT has been reported as orally active at a 15–20 mg dose, with its profound psychedelic effects beginning within 15 minutes and lasting about 2–3 h (Shulgin & Shulgin, 2017 ▸).

4-HO-DiPT glutarate, a ‘hemiester’ prodrug of 4-HO-DiPT has been reported in the patent literature (Bryson, 2022 ▸). We have previously published work characterizing tryptamine compounds, highlighting the importance of single-crystal X-ray diffraction studies when characterizing tryptamine salts because they can occur in a variety of forms that are often not appreciated by other means of characterization (Chadeayne *et al.*, 2019*a* ▸,*b* ▸; Chadeayne, Pham, Golen *et al.*, 2020 ▸; Sammeta *et al.*, 2020 ▸; Pham *et al.*, 2021 ▸; Naeem *et al.*, 2022 ▸). To this end, we synthesized 4-glutarato-*N*,*N*-diiso­propyl­trypamine and report herein two crystalline forms of the compound as both its methanol and ethanol solvates. 
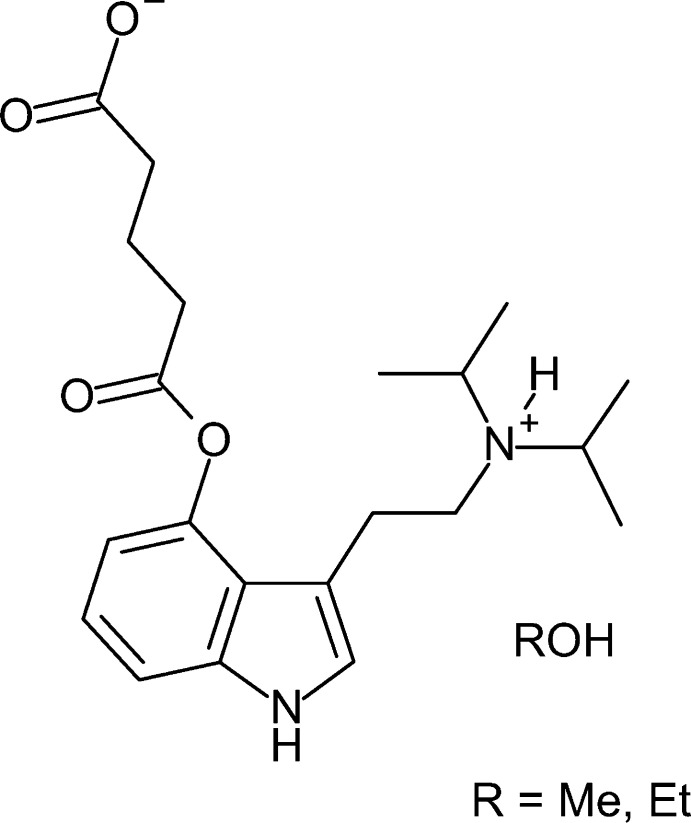


## 2. Structural commentary

**Figure 1:**
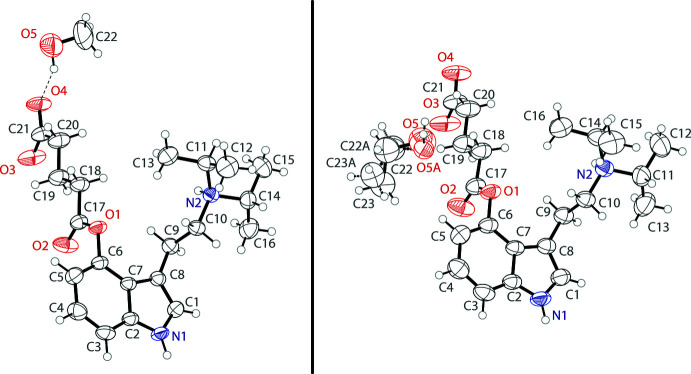
The mol­ecular structures of 4-glutarato-*N*,*N*-diiso­propyl­tryptamine as both its methanol (left) and ethanol (right) solvate, showing atomic labeling. Displacement ellipsoids are drawn at the 50% probability level. Hydrogen bonds are shown as dashed lines. Dashed bonds indicate the minor occupancy disordered component in the ethanol solvate.

In the solid state, the compound exists as a zwitterion, with a protonated tertiary ammonium group and a deprotonated carboxyl­ate of the glutarato group. Both of the solvate structures possess one zwitterionic mol­ecule and one alcohol mol­ecule in the asymmetric unit (Fig. 1 ▸). In the ethanol solvate, the alcohol mol­ecule is disordered over two orientations in a 0.531 (11):0.469 (11) ratio. Both solvates have near planar indole units with r.m.s. deviations from planarity of 0.009 and 0.016 Å for the methanol and ethanol solvates, respectively. The glutarato units are also close to planar with r.m.s. deviations of only 0.061 and 0.071 Å. In both cases, the glutarato unit is nearly orthogonal to the indole plane, showing plane-to-plane twists of 90.99 (6) and 94.21 (8)°. Likewise, the ethyl­amino arms are nearly orthogonal to the indole plane with C7—C8—C9—C10 angles of 90.2 (2) and 86.1 (3)°. Both ethyl­amino arms demonstrate *anti* configurations, with C8—C9—C10—N2 angles of 179.92 (14) and 180.0 (2)°. In both structures, the glutarato and ethyl­amino arms are turned to opposite sides of the indole. This differs from the structures observed in other zwitterionic indoles where intra­molecular hydrogen bonding leads to two groups being on the same side of the aromatic rings (Naeem *et al.*, 2022 ▸). The nature of the groups in this compound only allows for inter­molecular inter­actions (*vide infra*) and having the groups on opposite sides of the indole is sterically preferred.

## 3. Supra­molecular features

**Figure 2:**
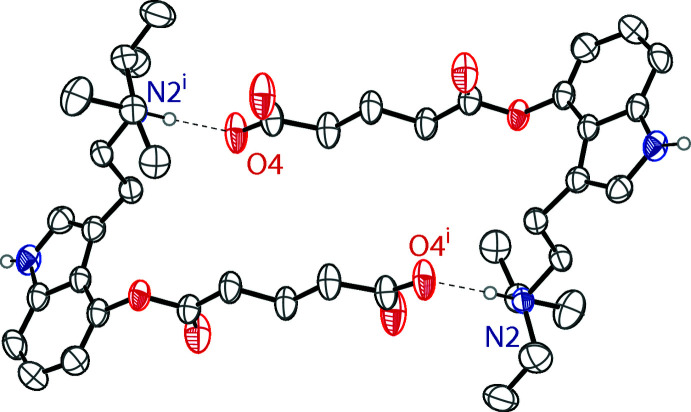
The ring formed by the dimerization of two zwitterionic 4-glutarato-*N*,*N*-diiso­propyl­tryptamine mol­ecules with graph set notation of 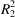 (28). The image shown is from the methanol solvate. Hydrogen bonds are shown as dashed lines. H atoms not involved in hydrogen bonding are omitted for clarity. Symmetry code: (i) 1 − *x*, 1 − *y*, 1 − *z*.

**Figure 3:**
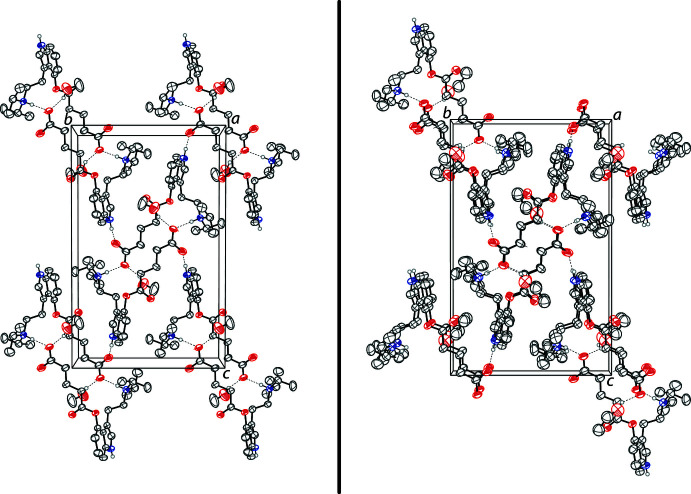
The crystal packing of the methanol solvate (left) and the ethanol solvate (right) of 4-glutarato-*N*,*N*-diiso­propyl­tryptamine, both shown along the *a*-axis. Hydrogen bonds are shown as dashed lines. H atoms not involved in hydrogen bonding are omitted for clarity.

In both crystals, the zwitterionic mol­ecules and alcohol solvents are held together by N^+^—H⋯O^−^ and O—H⋯O hydrogen bonds that produce infinite two-dimensional networks parallel to the (100) plane. The most significant hydrogen bonds are N2—H2⋯O4 bonds between the diiso­propyl­tryptammonium cation and the carboxyl­ate anion of another zwitterionic mol­ecule. These inter­actions form centrosymmetrical dimers, which form rings with graph-set notation of 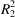 (28) (Etter *et al.*, 1990 ▸). These dimers are shown in Fig. 2 ▸. The dimers are joined together through N1—H1⋯O3 hydrogen bonds between the indole nitro­gen and the other carboxyl­ate oxygen. The alcohol oxygens also hydrogen bond to the carboxyl­ate anion through O5—H5⋯O4 bonds (Tables 1 ▸ and 2 ▸). The two structures demonstrate near identical hydrogen-bonding networks in the solid state, which can be seen in their packing diagrams (Fig. 3 ▸).

## 4. Database survey

There are three reported tryptamine structures possessing isopropyl groups on the ethyl­amino arm, all of which are *N*-methyl-*N*-isopropyl derivatives: *N*-methyl-*N*-iso­propyl­tryptammonium hydro­fumarate (Chadeayne, Pham, Golen *et al.*, 2019 ▸: RONSOF) as well as the hydro­fumarate (Chad­eayne, Pham, Golen *et al.*, 2019 ▸: RONSUL) and fumarate (Chadeayne, Pham, Golen *et al.*, 2020 ▸: TUFQAP) of 4-hy­droxy-*N*-methyl-*N*-iso­propyl­tryptamine. There are six structures of 4-substituted esters of tryptamines in the literature, all of which are 4-acet­oxy derivatives: the hydro­fumarate (Chadeayne *et al.*, 2019*a* ▸: HOCJUH) and fumarate (Chad­eayne *et al.*, 2019*b* ▸: XOFDOO) of psilacetin (4-acet­oxy-*N*,*N*-di­methyl­tryptamine), 4-acet­oxy-*N*-methyl-*N*-ethyl­trypt­ammo­nium hydro­fumarate (Pham *et al.*, 2021 ▸: OJIQIK), 4-acet­oxy-*N*-methyl-*N*-allyl­tryptammonium hydro­fumarate (Pham *et al.*, 2021 ▸: OJIQOQ), 4-acet­oxy-*N*,*N*-di­allyl­tryptammonium fumarate fumaric acid (Pham *et al.*, 2021 ▸: OJIQUW), and 4-acet­oxy-*N*,*N*,*N*-tri­methyl­tryptammonium iodide (Chadeayne, Pham, Reid *et al.*, 2020 ▸: XUXDUS). There are two tryptamine zwitterions reported in the literature, those being the natural products baeocystin, 4-phosphor­yloxy-*N*-methyl­tryptamine (Naeem *et al.*, 2022 ▸), and psilocybin, 4-phosphor­yloxy-*N*,*N*-di­methyl­tryptamine (Weber & Petcher, 1974 ▸; PSILOC; Sherwood *et al.*, 2022 ▸: TAVZID, TAVZID01; Greenan *et al.*, 2020 ▸; OKOKAD).

## 5. Synthesis and crystallization

112 mg of 4-hy­droxy-*N*,*N*-diiso­propyl­tryptamine (1 mmol) were dissolved in 5 mL of chloro­form. 0.3 mL of tri­ethyl­amine (5 mmol) followed by 490 mg of glutaric anhydride (10 mmol) were then added to the solution. The mixture was stirred at room temperature for 30 minutes, resulting in a precipitate which was isolated *via* filtration. The precipitate was triturated with tetra­hydro­furan and washed with chloro­form to obtain 73 mg of white powder (65% yield).

^1^H NMR (400 MHz, DMSO-*d*_6_): δ 11.02 (*s*, 1H, N*H*), 7.22 (*d*, *J* = 8.1 Hz, 1H, Ar*H*), 7.16 (*d*, *J* = 2.3 Hz, 1H, Ar*H*), 7.02 (*t*, *J* = 7.9 Hz, 1H, Ar*H*), 6.64 (*d*, *J* = 7.5 Hz, 1H, Ar*H*), 3.10 (*sept*, *J* = 6.5 Hz, 2H, C*H*), 2.77–2.63 (*m*, 6H, C*H*_2_), 2.31 (*t*, *J* = 7.2 Hz, 2H, C*H*_2_), 1.88 (*t*, *J* = 7.2 Hz, 2H, C*H*_2_), 1.00 (*d*, *J* = 6.6 Hz, 12H, C*H*_3_).

The powder was recrystallized from boiling methanol to yield single crystals of the methanol solvate suitable for X-ray diffraction analysis. Slow evaporation of an ethanol solution of the powder produced single crystals of the ethanol solvate suitable for X-ray diffraction analysis.

## 6. Refinement

Crystal data, data collection and structure refinement details are summarized in Table 3 ▸. In the methanol solvate, hydrogen atoms H1, H2 and H5*A* were found in a difference-Fourier map and in the ethanol solvate, hydrogen atoms H1 and H2 were found in a difference-Fourier map. These hydrogens were refined isotropically, using DFIX restraints with N—H(indole) distances of 0.87 (1) Å, N—H(ammonium) distances of 0.90 (1) Å, and O—H distances of 0.99 (1) Å. Isotropic displacement parameters were set to 1.2*U*_eq_ of the parent nitro­gen atoms and 1.5*U*_eq_ of the parent oxygen atom. All other hydrogens were placed in calculated positions [C—H = 0.93 Å (*sp* ^2^), 0.97 Å (CH_2_), 0.96 Å (CH_3_)]. The hydrogen atoms in the disordered ethanol mol­ecule were placed in calculated positions [O—H = 0.82 Å]. Isotropic displacement parameters were set to 1.2*U*_eq_ of the parent carbon atoms and 1.5*U*_eq_ of the parent oxygen atoms.

## Supplementary Material

Crystal structure: contains datablock(s) I, II, global. DOI: 10.1107/S2056989022009094/zn2023sup1.cif

Structure factors: contains datablock(s) I. DOI: 10.1107/S2056989022009094/zn2023Isup2.hkl

Structure factors: contains datablock(s) II. DOI: 10.1107/S2056989022009094/zn2023IIsup3.hkl

CCDC references: 2206880, 2206879

Additional supporting information:  crystallographic information; 3D view; checkCIF report

## Figures and Tables

**Table 1:** Hydrogen-bond geometry (Å, °) for the methanol solvate[Chem scheme1]

*D*—H⋯*A*	*D*—H	H⋯*A*	*D*⋯*A*	*D*—H⋯*A*
O5—H5*A*⋯O4	1.00 (1)	1.83 (2)	2.748 (2)	151 (3)
N2—H2⋯O4^i^	0.90 (1)	1.81 (1)	2.7154 (19)	177 (2)
N1—H1⋯O3^ii^	0.86 (1)	1.99 (1)	2.773 (2)	151 (2)

Symmetry codes: (i) 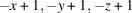 ; (ii) 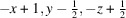 .

**Table 2:** Hydrogen-bond geometry (Å, °) for the ethanol solvate[Chem scheme1]

*D*—H⋯*A*	*D*—H	H⋯*A*	*D*⋯*A*	*D*—H⋯*A*
N2—H2⋯O4^i^	0.90 (3)	1.79 (3)	2.686 (3)	177 (3)
N1—H1⋯O3^ii^	0.85 (1)	1.91 (1)	2.751 (3)	167 (3)
O5—H5*A*⋯O4^iii^	0.82	1.97	2.692 (10)	147
O5*A*—H5*AA*⋯O4^iii^	0.82	1.95	2.732 (6)	160

Symmetry codes: (i) 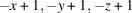 ; (ii) 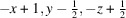 ; (iii) 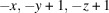 .

**Table 3:** Experimental details

	Methanol solvate	Ethanol solvate
Crystal data		
Chemical formula	C_21_H_30_N_2_O_4_·CH_4_O	C_21_H_30_N_2_O_4_·C_2_H_6_O
*M*_r_	406.51	420.54
Crystal system, space group	Monoclinic, *P*2_1_/*c*	Monoclinic, *P*2_1_/*c*
Temperature (K)	297	297
*a*, *b*, *c* (Å)	7.9531 (5), 13.4224 (7), 21.2015 (11)	8.0087 (12), 13.7968 (17), 21.878 (3)
β (°)	92.484 (2)	90.749 (4)
*V* (Å^3^)	2261.1 (2)	2417.2 (5)
*Z*	4	4
Radiation type	Mo *K*α	Mo *K*α
μ (mm^−1^)	0.08	0.08
Crystal size (mm)	0.22 × 0.21 × 0.20	0.30 × 0.27 × 0.22

Data collection		
Diffractometer	Bruker D8 Venture CMOS	Bruker D8 Venture CMOS
Absorption correction	Multi-scan (*SADABS*; Bruker, 2021 ▸)	Multi-scan (*SADABS*; Bruker, 2021 ▸)
*T*_min_, *T*_max_	0.718, 0.745	0.692, 0.745
No. of measured, independent and observed [*I* > 2σ(*I*)] reflections	61210, 4304, 3531	37412, 4461, 3038
*R*_int_	0.039	0.055
(sin θ/λ)_max_ (Å^−1^)	0.610	0.604

Refinement		
*R*[*F* ^2^ > 2σ(*F* ^2^)], *wR*(*F* ^2^), *S*	0.050, 0.143, 1.03	0.060, 0.176, 1.04
No. of reflections	4304	4461
No. of parameters	279	313
No. of restraints	3	46
H-atom treatment	H atoms treated by a mixture of independent and constrained refinement	H atoms treated by a mixture of independent and constrained refinement
Δρ_max_, Δρ_min_ (e Å^−3^)	0.46, −0.39	0.35, −0.46

Computer programs: *APEX4* and *SAINT* (Bruker, 2021 ▸), *SHELXT2014* (Sheldrick, 2015*a* ▸), *SHELXL2018* (Sheldrick, 2015*b* ▸), *OLEX2* (Dolomanov *et al.*, 2009 ▸), and *publCIF* (Westrip, 2010 ▸).

## supplementary crystallographic information

### 5-[(3-{2-[Bis(propan-2-yl)azaniumyl]ethyl}-1H-indol-4-yl)oxy]-5-oxopentanoate methanol monosolvate (I) Crystal data

**Table:**

C_21_H_30_N_2_O_4_·CH_4_O	*F*(000) = 880
*M**_r_* = 406.51	*D*_x_ = 1.194 Mg m^−^^3^
Monoclinic, *P*2_1_/*c*	Mo *K*α radiation, λ = 0.71073 Å
*a* = 7.9531 (5) Å	Cell parameters from 9859 reflections
*b* = 13.4224 (7) Å	θ = 3.0–25.6°
*c* = 21.2015 (11) Å	µ = 0.08 mm^−^^1^
β = 92.484 (2)°	*T* = 297 K
*V* = 2261.1 (2) Å^3^	Block, colourless
*Z* = 4	0.22 × 0.21 × 0.20 mm

### 5-[(3-{2-[Bis(propan-2-yl)azaniumyl]ethyl}-1H-indol-4-yl)oxy]-5-oxopentanoate methanol monosolvate (I) Data collection

**Table:**

Bruker D8 Venture CMOS diffractometer	3531 reflections with *I* > 2σ(*I*)
φ and ω scans	*R*_int_ = 0.039
Absorption correction: multi-scan (SADABS; Bruker, 2021)	θ_max_ = 25.7°, θ_min_ = 3.0°
*T*_min_ = 0.718, *T*_max_ = 0.745	*h* = −9→9
61210 measured reflections	*k* = −16→16
4304 independent reflections	*l* = −25→25

### 5-[(3-{2-[Bis(propan-2-yl)azaniumyl]ethyl}-1H-indol-4-yl)oxy]-5-oxopentanoate methanol monosolvate (I) Refinement

**Table:**

Refinement on *F*^2^	Hydrogen site location: mixed
Least-squares matrix: full	H atoms treated by a mixture of independent and constrained refinement
*R*[*F*^2^ > 2σ(*F*^2^)] = 0.050	*w* = 1/[σ^2^(*F*_o_^2^) + (0.0677*P*)^2^ + 1.0658*P*] where *P* = (*F*_o_^2^ + 2*F*_c_^2^)/3
*wR*(*F*^2^) = 0.143	(Δ/σ)_max_ < 0.001
*S* = 1.03	Δρ_max_ = 0.46 e Å^−^^3^
4304 reflections	Δρ_min_ = −0.39 e Å^−^^3^
279 parameters	Extinction correction: SHELXL2018 (Sheldrick, 2015b), Fc^*^=kFc[1+0.001xFc^2^λ^3^/sin(2θ)]^-1/4^
3 restraints	Extinction coefficient: 0.0049 (16)

### 5-[(3-{2-[Bis(propan-2-yl)azaniumyl]ethyl}-1H-indol-4-yl)oxy]-5-oxopentanoate methanol monosolvate (I) Special details

**Table:**

Geometry. All esds (except the esd in the dihedral angle between two l.s. planes) are estimated using the full covariance matrix. The cell esds are taken into account individually in the estimation of esds in distances, angles and torsion angles; correlations between esds in cell parameters are only used when they are defined by crystal symmetry. An approximate (isotropic) treatment of cell esds is used for estimating esds involving l.s. planes.

### 5-[(3-{2-[Bis(propan-2-yl)azaniumyl]ethyl}-1H-indol-4-yl)oxy]-5-oxopentanoate methanol monosolvate (I) Fractional atomic coordinates and isotropic or equivalent isotropic displacement parameters (Å^2^)

**Table:**

	*x*	*y*	*z*	*U*_iso_*/*U*_eq_	
O1	0.17552 (17)	0.35382 (9)	0.29846 (5)	0.0421 (3)	
O2	0.2385 (2)	0.51443 (11)	0.28621 (6)	0.0675 (5)	
O3	0.3521 (2)	0.72404 (12)	0.48361 (7)	0.0730 (5)	
O4	0.2931 (2)	0.66404 (11)	0.57648 (6)	0.0621 (4)	
N1	0.4481 (2)	0.26115 (12)	0.11776 (7)	0.0454 (4)	
N2	0.61740 (18)	0.15461 (10)	0.37628 (6)	0.0362 (3)	
C1	0.5573 (2)	0.25778 (14)	0.16893 (9)	0.0446 (4)	
H1A	0.669647	0.238884	0.167694	0.053*	
C2	0.2940 (2)	0.29148 (13)	0.13645 (8)	0.0386 (4)	
C3	0.1426 (3)	0.30411 (15)	0.10225 (8)	0.0481 (5)	
H3	0.134321	0.291932	0.059041	0.058*	
C4	0.0058 (3)	0.33510 (17)	0.13421 (9)	0.0539 (5)	
H4	−0.096839	0.343720	0.112189	0.065*	
C5	0.0169 (2)	0.35412 (15)	0.19936 (9)	0.0489 (5)	
H5	−0.077222	0.375904	0.219944	0.059*	
C6	0.1662 (2)	0.34057 (12)	0.23242 (8)	0.0375 (4)	
C7	0.3099 (2)	0.30850 (12)	0.20252 (7)	0.0348 (4)	
C8	0.4801 (2)	0.28580 (12)	0.22217 (8)	0.0381 (4)	
C9	0.5583 (2)	0.27857 (13)	0.28772 (8)	0.0410 (4)	
H9A	0.501378	0.323224	0.315755	0.049*	
H9B	0.675960	0.297597	0.287599	0.049*	
C10	0.5426 (2)	0.17173 (13)	0.31047 (8)	0.0387 (4)	
H10A	0.424472	0.153645	0.309562	0.046*	
H10B	0.598183	0.128085	0.281434	0.046*	
C11	0.4906 (2)	0.10852 (14)	0.41929 (9)	0.0452 (4)	
H11	0.547211	0.100130	0.460941	0.054*	
C13	0.3444 (3)	0.17846 (17)	0.42725 (11)	0.0581 (5)	
H13A	0.385521	0.241480	0.442784	0.087*	
H13B	0.269327	0.150484	0.456783	0.087*	
H13C	0.285274	0.187901	0.387253	0.087*	
C12	0.4347 (4)	0.00616 (17)	0.39679 (13)	0.0723 (7)	
H12A	0.530499	−0.037275	0.396077	0.108*	
H12B	0.383512	0.011193	0.355068	0.108*	
H12C	0.354637	−0.020317	0.424972	0.108*	
C14	0.7823 (2)	0.09675 (15)	0.37460 (9)	0.0471 (5)	
H14	0.759363	0.032539	0.353974	0.057*	
C15	0.8546 (3)	0.0769 (2)	0.44071 (11)	0.0712 (7)	
H15A	0.783385	0.030922	0.461678	0.107*	
H15B	0.861037	0.138280	0.463944	0.107*	
H15C	0.965311	0.048954	0.438396	0.107*	
C16	0.9078 (3)	0.15321 (19)	0.33620 (12)	0.0632 (6)	
H16A	0.862701	0.161671	0.293803	0.095*	
H16B	1.011172	0.116333	0.335554	0.095*	
H16C	0.929013	0.217350	0.354941	0.095*	
C17	0.2114 (2)	0.44694 (13)	0.32012 (8)	0.0409 (4)	
C18	0.2177 (3)	0.44943 (14)	0.39084 (8)	0.0483 (5)	
H18A	0.306025	0.404959	0.406611	0.058*	
H18B	0.111785	0.424768	0.405632	0.058*	
C19	0.2497 (3)	0.55192 (15)	0.41772 (9)	0.0522 (5)	
H19A	0.162315	0.596552	0.401363	0.063*	
H19B	0.356367	0.576115	0.403349	0.063*	
C20	0.2538 (3)	0.55546 (15)	0.48899 (9)	0.0536 (5)	
H20A	0.332027	0.505235	0.505171	0.064*	
H20B	0.143059	0.538020	0.502980	0.064*	
C21	0.3041 (3)	0.65529 (15)	0.51767 (9)	0.0474 (5)	
O5	0.1577 (3)	0.53742 (16)	0.66260 (11)	0.0928 (6)	
H5A	0.224 (4)	0.563 (2)	0.6271 (11)	0.111*	
C22	0.2529 (6)	0.4611 (3)	0.6828 (2)	0.1272 (15)	
H22A	0.184260	0.413329	0.703499	0.191*	
H22B	0.339437	0.484832	0.712036	0.191*	
H22C	0.303585	0.430125	0.647521	0.191*	
H2	0.644 (2)	0.2153 (9)	0.3923 (9)	0.045 (5)*	
H1	0.475 (3)	0.2488 (16)	0.0796 (6)	0.057 (6)*	

### 5-[(3-{2-[Bis(propan-2-yl)azaniumyl]ethyl}-1H-indol-4-yl)oxy]-5-oxopentanoate methanol monosolvate (I) Atomic displacement parameters (Å^2^)

**Table:**

	*U*^11^	*U*^22^	*U*^33^	*U*^12^	*U*^13^	*U*^23^
O1	0.0613 (8)	0.0378 (6)	0.0278 (6)	−0.0046 (6)	0.0104 (5)	−0.0059 (5)
O2	0.1172 (14)	0.0483 (8)	0.0375 (7)	−0.0279 (9)	0.0073 (8)	0.0011 (6)
O3	0.1127 (14)	0.0647 (10)	0.0436 (8)	−0.0400 (9)	0.0275 (8)	−0.0135 (7)
O4	0.1033 (12)	0.0526 (8)	0.0311 (7)	−0.0267 (8)	0.0110 (7)	−0.0100 (6)
N1	0.0607 (10)	0.0477 (9)	0.0289 (7)	0.0035 (7)	0.0133 (7)	−0.0045 (6)
N2	0.0427 (8)	0.0336 (7)	0.0321 (7)	−0.0030 (6)	−0.0009 (6)	−0.0024 (6)
C1	0.0484 (10)	0.0436 (10)	0.0421 (10)	0.0040 (8)	0.0068 (8)	−0.0017 (8)
C2	0.0537 (10)	0.0338 (8)	0.0286 (8)	−0.0027 (7)	0.0058 (7)	−0.0025 (6)
C3	0.0624 (12)	0.0538 (11)	0.0278 (8)	−0.0041 (9)	−0.0028 (8)	−0.0026 (8)
C4	0.0502 (11)	0.0655 (13)	0.0452 (11)	−0.0023 (10)	−0.0074 (9)	0.0017 (9)
C5	0.0455 (10)	0.0558 (11)	0.0460 (11)	0.0010 (9)	0.0075 (8)	−0.0030 (9)
C6	0.0500 (10)	0.0347 (8)	0.0283 (8)	−0.0044 (7)	0.0073 (7)	−0.0033 (6)
C7	0.0476 (9)	0.0301 (8)	0.0266 (8)	−0.0030 (7)	0.0026 (7)	−0.0017 (6)
C8	0.0480 (10)	0.0333 (8)	0.0331 (8)	−0.0006 (7)	0.0028 (7)	−0.0002 (7)
C9	0.0477 (10)	0.0379 (9)	0.0370 (9)	−0.0015 (7)	−0.0027 (7)	−0.0032 (7)
C10	0.0440 (9)	0.0398 (9)	0.0318 (8)	−0.0042 (7)	−0.0027 (7)	−0.0033 (7)
C11	0.0531 (11)	0.0423 (10)	0.0404 (9)	−0.0060 (8)	0.0054 (8)	0.0046 (8)
C13	0.0523 (12)	0.0638 (13)	0.0592 (13)	−0.0052 (10)	0.0121 (10)	−0.0035 (10)
C12	0.0905 (18)	0.0434 (12)	0.0845 (17)	−0.0185 (12)	0.0207 (14)	0.0013 (11)
C14	0.0463 (10)	0.0472 (10)	0.0475 (10)	0.0064 (8)	−0.0030 (8)	−0.0041 (8)
C15	0.0655 (15)	0.0859 (17)	0.0609 (14)	0.0185 (13)	−0.0131 (11)	0.0064 (12)
C16	0.0448 (11)	0.0772 (16)	0.0678 (14)	0.0022 (10)	0.0058 (10)	−0.0027 (12)
C17	0.0503 (10)	0.0382 (9)	0.0349 (9)	−0.0052 (8)	0.0075 (7)	−0.0045 (7)
C18	0.0719 (13)	0.0414 (10)	0.0319 (9)	−0.0056 (9)	0.0054 (8)	−0.0059 (7)
C19	0.0747 (14)	0.0454 (11)	0.0367 (10)	−0.0082 (9)	0.0036 (9)	−0.0102 (8)
C20	0.0731 (14)	0.0505 (11)	0.0380 (10)	−0.0169 (10)	0.0104 (9)	−0.0105 (8)
C21	0.0573 (11)	0.0509 (11)	0.0349 (9)	−0.0159 (9)	0.0107 (8)	−0.0100 (8)
O5	0.0849 (13)	0.0864 (14)	0.1090 (16)	0.0042 (11)	0.0245 (11)	0.0304 (12)
C22	0.152 (4)	0.073 (2)	0.159 (4)	0.045 (2)	0.036 (3)	0.038 (2)

### 5-[(3-{2-[Bis(propan-2-yl)azaniumyl]ethyl}-1H-indol-4-yl)oxy]-5-oxopentanoate methanol monosolvate (I) Geometric parameters (Å, º)

**Table:**

O1—C6	1.4100 (19)	C11—C12	1.515 (3)
O1—C17	1.358 (2)	C13—H13A	0.9600
O2—C17	1.182 (2)	C13—H13B	0.9600
O3—C21	1.242 (2)	C13—H13C	0.9600
O4—C21	1.259 (2)	C12—H12A	0.9600
N1—C1	1.361 (2)	C12—H12B	0.9600
N1—C2	1.366 (2)	C12—H12C	0.9600
N1—H1	0.862 (9)	C14—H14	0.9800
N2—C10	1.510 (2)	C14—C15	1.515 (3)
N2—C11	1.520 (2)	C14—C16	1.518 (3)
N2—C14	1.526 (2)	C15—H15A	0.9600
N2—H2	0.903 (9)	C15—H15B	0.9600
C1—H1A	0.9300	C15—H15C	0.9600
C1—C8	1.361 (2)	C16—H16A	0.9600
C2—C3	1.389 (3)	C16—H16B	0.9600
C2—C7	1.420 (2)	C16—H16C	0.9600
C3—H3	0.9300	C17—C18	1.498 (2)
C3—C4	1.371 (3)	C18—H18A	0.9700
C4—H4	0.9300	C18—H18B	0.9700
C4—C5	1.404 (3)	C18—C19	1.506 (3)
C5—H5	0.9300	C19—H19A	0.9700
C5—C6	1.364 (3)	C19—H19B	0.9700
C6—C7	1.399 (2)	C19—C20	1.511 (3)
C7—C8	1.431 (3)	C20—H20A	0.9700
C8—C9	1.501 (2)	C20—H20B	0.9700
C9—H9A	0.9700	C20—C21	1.518 (3)
C9—H9B	0.9700	O5—H5A	0.998 (10)
C9—C10	1.520 (2)	O5—C22	1.334 (4)
C10—H10A	0.9700	C22—H22A	0.9600
C10—H10B	0.9700	C22—H22B	0.9600
C11—H11	0.9800	C22—H22C	0.9600
C11—C13	1.510 (3)		
			
C17—O1—C6	116.95 (13)	H13B—C13—H13C	109.5
C1—N1—C2	109.15 (14)	C11—C12—H12A	109.5
C1—N1—H1	124.4 (15)	C11—C12—H12B	109.5
C2—N1—H1	126.4 (15)	C11—C12—H12C	109.5
C10—N2—C11	111.80 (14)	H12A—C12—H12B	109.5
C10—N2—C14	111.11 (13)	H12A—C12—H12C	109.5
C10—N2—H2	106.6 (13)	H12B—C12—H12C	109.5
C11—N2—C14	113.58 (14)	N2—C14—H14	108.3
C11—N2—H2	106.8 (13)	C15—C14—N2	111.09 (16)
C14—N2—H2	106.4 (13)	C15—C14—H14	108.3
N1—C1—H1A	124.6	C15—C14—C16	110.61 (19)
N1—C1—C8	110.89 (17)	C16—C14—N2	110.14 (16)
C8—C1—H1A	124.6	C16—C14—H14	108.3
N1—C2—C3	130.79 (16)	C14—C15—H15A	109.5
N1—C2—C7	106.95 (15)	C14—C15—H15B	109.5
C3—C2—C7	122.25 (17)	C14—C15—H15C	109.5
C2—C3—H3	121.1	H15A—C15—H15B	109.5
C4—C3—C2	117.88 (17)	H15A—C15—H15C	109.5
C4—C3—H3	121.1	H15B—C15—H15C	109.5
C3—C4—H4	119.2	C14—C16—H16A	109.5
C3—C4—C5	121.60 (18)	C14—C16—H16B	109.5
C5—C4—H4	119.2	C14—C16—H16C	109.5
C4—C5—H5	120.1	H16A—C16—H16B	109.5
C6—C5—C4	119.84 (18)	H16A—C16—H16C	109.5
C6—C5—H5	120.1	H16B—C16—H16C	109.5
C5—C6—O1	120.02 (16)	O1—C17—C18	110.89 (14)
C5—C6—C7	121.21 (16)	O2—C17—O1	122.74 (16)
C7—C6—O1	118.69 (15)	O2—C17—C18	126.32 (17)
C2—C7—C8	107.19 (15)	C17—C18—H18A	108.9
C6—C7—C2	117.21 (16)	C17—C18—H18B	108.9
C6—C7—C8	135.60 (15)	C17—C18—C19	113.38 (16)
C1—C8—C7	105.81 (15)	H18A—C18—H18B	107.7
C1—C8—C9	124.48 (17)	C19—C18—H18A	108.9
C7—C8—C9	129.19 (15)	C19—C18—H18B	108.9
C8—C9—H9A	110.0	C18—C19—H19A	108.8
C8—C9—H9B	110.0	C18—C19—H19B	108.8
C8—C9—C10	108.57 (14)	C18—C19—C20	113.78 (16)
H9A—C9—H9B	108.4	H19A—C19—H19B	107.7
C10—C9—H9A	110.0	C20—C19—H19A	108.8
C10—C9—H9B	110.0	C20—C19—H19B	108.8
N2—C10—C9	113.72 (13)	C19—C20—H20A	108.5
N2—C10—H10A	108.8	C19—C20—H20B	108.5
N2—C10—H10B	108.8	C19—C20—C21	114.98 (16)
C9—C10—H10A	108.8	H20A—C20—H20B	107.5
C9—C10—H10B	108.8	C21—C20—H20A	108.5
H10A—C10—H10B	107.7	C21—C20—H20B	108.5
N2—C11—H11	107.3	O3—C21—O4	122.88 (17)
C13—C11—N2	110.38 (15)	O3—C21—C20	120.35 (16)
C13—C11—H11	107.3	O4—C21—C20	116.77 (17)
C13—C11—C12	112.46 (19)	C22—O5—H5A	101 (2)
C12—C11—N2	111.93 (16)	O5—C22—H22A	109.5
C12—C11—H11	107.3	O5—C22—H22B	109.5
C11—C13—H13A	109.5	O5—C22—H22C	109.5
C11—C13—H13B	109.5	H22A—C22—H22B	109.5
C11—C13—H13C	109.5	H22A—C22—H22C	109.5
H13A—C13—H13B	109.5	H22B—C22—H22C	109.5
H13A—C13—H13C	109.5		
			
O1—C6—C7—C2	177.15 (14)	C6—O1—C17—O2	−2.0 (3)
O1—C6—C7—C8	−2.1 (3)	C6—O1—C17—C18	−179.77 (15)
O1—C17—C18—C19	−177.50 (17)	C6—C7—C8—C1	179.74 (19)
O2—C17—C18—C19	4.8 (3)	C6—C7—C8—C9	7.9 (3)
N1—C1—C8—C7	−0.1 (2)	C7—C2—C3—C4	0.7 (3)
N1—C1—C8—C9	172.23 (16)	C7—C8—C9—C10	90.2 (2)
N1—C2—C3—C4	179.42 (19)	C8—C9—C10—N2	179.92 (14)
N1—C2—C7—C6	179.94 (15)	C10—N2—C11—C13	−63.01 (19)
N1—C2—C7—C8	−0.61 (19)	C10—N2—C11—C12	63.1 (2)
C1—N1—C2—C3	−178.34 (19)	C10—N2—C14—C15	−178.31 (17)
C1—N1—C2—C7	0.5 (2)	C10—N2—C14—C16	58.79 (19)
C1—C8—C9—C10	−80.3 (2)	C11—N2—C10—C9	125.26 (16)
C2—N1—C1—C8	−0.3 (2)	C11—N2—C14—C15	−51.2 (2)
C2—C3—C4—C5	0.3 (3)	C11—N2—C14—C16	−174.13 (16)
C2—C7—C8—C1	0.45 (19)	C14—N2—C10—C9	−106.70 (17)
C2—C7—C8—C9	−171.42 (16)	C14—N2—C11—C13	170.27 (16)
C3—C2—C7—C6	−1.1 (2)	C14—N2—C11—C12	−63.6 (2)
C3—C2—C7—C8	178.39 (16)	C17—O1—C6—C5	−89.2 (2)
C3—C4—C5—C6	−0.8 (3)	C17—O1—C6—C7	94.06 (19)
C4—C5—C6—O1	−176.20 (17)	C17—C18—C19—C20	179.16 (18)
C4—C5—C6—C7	0.4 (3)	C18—C19—C20—C21	173.75 (19)
C5—C6—C7—C2	0.5 (2)	C19—C20—C21—O3	−6.2 (3)
C5—C6—C7—C8	−178.76 (19)	C19—C20—C21—O4	173.2 (2)

### 5-[(3-{2-[Bis(propan-2-yl)azaniumyl]ethyl}-1H-indol-4-yl)oxy]-5-oxopentanoate methanol monosolvate (I) Hydrogen-bond geometry (Å, º)

**Table:**

*D*—H···*A*	*D*—H	H···*A*	*D*···*A*	*D*—H···*A*
O5—H5*A*···O4	1.00 (1)	1.83 (2)	2.748 (2)	151 (3)
N2—H2···O4^i^	0.90 (1)	1.81 (1)	2.7154 (19)	177 (2)
N1—H1···O3^ii^	0.86 (1)	1.99 (1)	2.773 (2)	151 (2)

Symmetry codes: (i) −*x*+1, −*y*+1, −*z*+1; (ii) −*x*+1, *y*−1/2, −*z*+1/2.

### 5-[(3-{2-[Bis(propan-2-yl)azaniumyl]ethyl}-1H-indol-4-yl)oxy]-5-oxopentanoate ethanol monosolvate (II) Crystal data

**Table:**

C_21_H_30_N_2_O_4_·C_2_H_6_O	*F*(000) = 912
*M**_r_* = 420.54	*D*_x_ = 1.156 Mg m^−^^3^
Monoclinic, *P*2_1_/*c*	Mo *K*α radiation, λ = 0.71073 Å
*a* = 8.0087 (12) Å	Cell parameters from 7678 reflections
*b* = 13.7968 (17) Å	θ = 2.5–24.9°
*c* = 21.878 (3) Å	µ = 0.08 mm^−^^1^
β = 90.749 (4)°	*T* = 297 K
*V* = 2417.2 (5) Å^3^	Block, colourless
*Z* = 4	0.30 × 0.27 × 0.22 mm

### 5-[(3-{2-[Bis(propan-2-yl)azaniumyl]ethyl}-1H-indol-4-yl)oxy]-5-oxopentanoate ethanol monosolvate (II) Data collection

**Table:**

Bruker D8 Venture CMOS diffractometer	3038 reflections with *I* > 2σ(*I*)
φ and ω scans	*R*_int_ = 0.055
Absorption correction: multi-scan (SADABS; Bruker, 2021)	θ_max_ = 25.4°, θ_min_ = 2.5°
*T*_min_ = 0.692, *T*_max_ = 0.745	*h* = −9→9
37412 measured reflections	*k* = −16→16
4461 independent reflections	*l* = −26→26

### 5-[(3-{2-[Bis(propan-2-yl)azaniumyl]ethyl}-1H-indol-4-yl)oxy]-5-oxopentanoate ethanol monosolvate (II) Refinement

**Table:**

Refinement on *F*^2^	46 restraints
Least-squares matrix: full	Hydrogen site location: mixed
*R*[*F*^2^ > 2σ(*F*^2^)] = 0.060	H atoms treated by a mixture of independent and constrained refinement
*wR*(*F*^2^) = 0.176	*w* = 1/[σ^2^(*F*_o_^2^) + (0.0772*P*)^2^ + 1.2353*P*] where *P* = (*F*_o_^2^ + 2*F*_c_^2^)/3
*S* = 1.04	(Δ/σ)_max_ < 0.001
4461 reflections	Δρ_max_ = 0.35 e Å^−^^3^
313 parameters	Δρ_min_ = −0.46 e Å^−^^3^

### 5-[(3-{2-[Bis(propan-2-yl)azaniumyl]ethyl}-1H-indol-4-yl)oxy]-5-oxopentanoate ethanol monosolvate (II) Special details

**Table:**

Geometry. All esds (except the esd in the dihedral angle between two l.s. planes) are estimated using the full covariance matrix. The cell esds are taken into account individually in the estimation of esds in distances, angles and torsion angles; correlations between esds in cell parameters are only used when they are defined by crystal symmetry. An approximate (isotropic) treatment of cell esds is used for estimating esds involving l.s. planes.

### 5-[(3-{2-[Bis(propan-2-yl)azaniumyl]ethyl}-1H-indol-4-yl)oxy]-5-oxopentanoate ethanol monosolvate (II) Fractional atomic coordinates and isotropic or equivalent isotropic displacement parameters (Å^2^)

**Table:**

	*x*	*y*	*z*	*U*_iso_*/*U*_eq_	Occ. (<1)
O1	0.2092 (2)	0.36531 (11)	0.30305 (7)	0.0553 (5)	
O2	0.2584 (3)	0.52278 (14)	0.28903 (8)	0.0832 (7)	
O3	0.3563 (3)	0.73209 (16)	0.48029 (8)	0.0872 (7)	
O4	0.2945 (3)	0.67092 (14)	0.56965 (8)	0.0787 (7)	
N1	0.4952 (3)	0.27474 (16)	0.12915 (9)	0.0595 (6)	
N2	0.6135 (3)	0.16000 (15)	0.37926 (9)	0.0508 (5)	
C1	0.5975 (4)	0.26944 (19)	0.17914 (11)	0.0583 (7)	
H1A	0.708687	0.250056	0.178280	0.070*	
C2	0.3413 (4)	0.30442 (17)	0.14673 (10)	0.0513 (6)	
C3	0.1947 (4)	0.31751 (19)	0.11340 (12)	0.0630 (7)	
H3	0.191217	0.307090	0.071410	0.076*	
C4	0.0551 (4)	0.3462 (2)	0.14408 (13)	0.0701 (8)	
H4	−0.044828	0.354362	0.122584	0.084*	
C5	0.0599 (4)	0.3635 (2)	0.20711 (12)	0.0628 (7)	
H5	−0.035912	0.383807	0.226972	0.075*	
C6	0.2045 (3)	0.35080 (17)	0.23928 (10)	0.0504 (6)	
C7	0.3503 (3)	0.31992 (15)	0.21087 (10)	0.0461 (6)	
C8	0.5159 (3)	0.29619 (16)	0.23053 (10)	0.0492 (6)	
C9	0.5832 (3)	0.28496 (17)	0.29440 (11)	0.0532 (6)	
H9A	0.528225	0.330228	0.321445	0.064*	
H9B	0.702044	0.298597	0.295389	0.064*	
C10	0.5518 (3)	0.18157 (17)	0.31541 (10)	0.0513 (6)	
H10A	0.432721	0.168963	0.313271	0.062*	
H10B	0.605720	0.137478	0.287333	0.062*	
C11	0.7716 (4)	0.0990 (2)	0.37851 (13)	0.0689 (8)	
H11	0.745795	0.037954	0.357516	0.083*	
C12	0.8324 (5)	0.0750 (3)	0.44274 (16)	0.0990 (12)	
H12A	0.753581	0.032798	0.461969	0.148*	
H12B	0.843001	0.133711	0.466050	0.148*	
H12C	0.938971	0.043428	0.440839	0.148*	
C13	0.9059 (4)	0.1506 (3)	0.34291 (17)	0.0903 (11)	
H13A	0.866622	0.162243	0.301912	0.135*	
H13B	1.004393	0.111058	0.341935	0.135*	
H13C	0.931685	0.211311	0.362305	0.135*	
C14	0.4773 (4)	0.11792 (19)	0.41877 (12)	0.0615 (7)	
H14	0.526311	0.106134	0.459368	0.074*	
C15	0.4148 (5)	0.0211 (2)	0.39449 (17)	0.0947 (12)	
H15A	0.505966	−0.023937	0.393021	0.142*	
H15B	0.368656	0.029758	0.354130	0.142*	
H15C	0.330151	−0.003624	0.420973	0.142*	
C16	0.3377 (4)	0.1903 (2)	0.42646 (14)	0.0753 (9)	
H16A	0.255515	0.164077	0.453397	0.113*	
H16B	0.286909	0.203677	0.387375	0.113*	
H16C	0.381923	0.249228	0.443505	0.113*	
C17	0.2386 (3)	0.45697 (18)	0.32284 (11)	0.0538 (6)	
C18	0.2450 (4)	0.46119 (18)	0.39101 (11)	0.0596 (7)	
H18A	0.336436	0.420965	0.405661	0.072*	
H18B	0.142255	0.434400	0.406826	0.072*	
C19	0.2678 (4)	0.56242 (19)	0.41582 (11)	0.0625 (7)	
H19A	0.373719	0.587837	0.402005	0.075*	
H19B	0.179984	0.603603	0.399319	0.075*	
C20	0.2642 (4)	0.5668 (2)	0.48422 (11)	0.0690 (8)	
H20A	0.341419	0.518749	0.500346	0.083*	
H20B	0.153216	0.548829	0.497359	0.083*	
C21	0.3081 (4)	0.6643 (2)	0.51231 (11)	0.0627 (7)	
H2	0.640 (3)	0.217 (2)	0.3959 (12)	0.064 (8)*	
H1	0.526 (3)	0.261 (2)	0.0929 (6)	0.068 (8)*	
O5	−0.1278 (12)	0.4643 (7)	0.3667 (5)	0.123 (3)	0.531 (11)
H5A	−0.165922	0.438097	0.397138	0.184*	0.531 (11)
C22	−0.2489 (13)	0.5309 (7)	0.3417 (5)	0.106 (3)	0.531 (11)
H22A	−0.281815	0.577081	0.372735	0.127*	0.531 (11)
H22B	−0.347635	0.495888	0.328047	0.127*	0.531 (11)
C23	−0.1720 (13)	0.5827 (6)	0.2891 (3)	0.106 (3)	0.531 (11)
H23A	−0.251356	0.627541	0.271841	0.159*	0.531 (11)
H23B	−0.074882	0.617423	0.303038	0.159*	0.531 (11)
H23C	−0.140469	0.536518	0.258509	0.159*	0.531 (11)
O5A	−0.1337 (9)	0.4474 (4)	0.3501 (3)	0.0655 (18)	0.469 (11)
H5AA	−0.198660	0.410977	0.367474	0.098*	0.469 (11)
C22A	−0.1963 (16)	0.5437 (6)	0.3520 (4)	0.098 (3)	0.469 (11)
H22C	−0.106576	0.590542	0.356016	0.117*	0.469 (11)
H22D	−0.273691	0.552244	0.385271	0.117*	0.469 (11)
C23A	−0.2814 (13)	0.5518 (7)	0.2923 (4)	0.097 (3)	0.469 (11)
H23D	−0.329689	0.615146	0.288185	0.146*	0.469 (11)
H23E	−0.202252	0.541673	0.260303	0.146*	0.469 (11)
H23F	−0.367862	0.503720	0.289294	0.146*	0.469 (11)

### 5-[(3-{2-[Bis(propan-2-yl)azaniumyl]ethyl}-1H-indol-4-yl)oxy]-5-oxopentanoate ethanol monosolvate (II) Atomic displacement parameters (Å^2^)

**Table:**

	*U*^11^	*U*^22^	*U*^33^	*U*^12^	*U*^13^	*U*^23^
O1	0.0813 (13)	0.0451 (9)	0.0399 (9)	−0.0016 (8)	0.0149 (8)	−0.0089 (7)
O2	0.142 (2)	0.0566 (12)	0.0510 (11)	−0.0251 (12)	0.0040 (12)	0.0006 (9)
O3	0.142 (2)	0.0749 (13)	0.0456 (10)	−0.0433 (13)	0.0284 (11)	−0.0115 (10)
O4	0.1303 (19)	0.0686 (12)	0.0376 (9)	−0.0367 (12)	0.0170 (10)	−0.0126 (8)
N1	0.0858 (17)	0.0567 (13)	0.0364 (11)	0.0033 (11)	0.0155 (11)	−0.0061 (9)
N2	0.0669 (14)	0.0427 (11)	0.0428 (11)	−0.0010 (10)	0.0009 (9)	−0.0050 (9)
C1	0.0704 (18)	0.0556 (15)	0.0491 (14)	0.0020 (13)	0.0113 (12)	−0.0018 (11)
C2	0.0757 (18)	0.0403 (12)	0.0382 (12)	−0.0019 (12)	0.0086 (12)	−0.0034 (10)
C3	0.090 (2)	0.0592 (16)	0.0391 (13)	−0.0040 (15)	−0.0046 (14)	−0.0045 (11)
C4	0.075 (2)	0.0740 (19)	0.0604 (17)	−0.0038 (16)	−0.0109 (15)	−0.0013 (14)
C5	0.0634 (18)	0.0629 (17)	0.0621 (16)	0.0023 (13)	0.0069 (14)	−0.0077 (13)
C6	0.0693 (17)	0.0428 (13)	0.0392 (12)	−0.0041 (11)	0.0078 (11)	−0.0071 (10)
C7	0.0662 (16)	0.0360 (11)	0.0360 (11)	−0.0054 (10)	0.0050 (10)	−0.0042 (9)
C8	0.0650 (16)	0.0410 (12)	0.0417 (12)	−0.0040 (11)	0.0050 (11)	−0.0024 (10)
C9	0.0671 (17)	0.0480 (14)	0.0444 (13)	−0.0043 (12)	0.0013 (11)	−0.0063 (10)
C10	0.0649 (16)	0.0482 (14)	0.0407 (12)	−0.0041 (12)	−0.0010 (11)	−0.0045 (10)
C11	0.078 (2)	0.0663 (18)	0.0619 (16)	0.0174 (15)	−0.0067 (14)	−0.0074 (14)
C12	0.103 (3)	0.113 (3)	0.080 (2)	0.033 (2)	−0.021 (2)	0.003 (2)
C13	0.066 (2)	0.112 (3)	0.094 (2)	0.0162 (19)	0.0070 (18)	−0.011 (2)
C14	0.081 (2)	0.0516 (14)	0.0520 (14)	−0.0085 (14)	0.0075 (13)	0.0042 (12)
C15	0.126 (3)	0.0562 (18)	0.102 (3)	−0.0246 (19)	0.022 (2)	0.0006 (17)
C16	0.078 (2)	0.076 (2)	0.0728 (19)	−0.0069 (16)	0.0166 (16)	−0.0036 (15)
C17	0.0651 (17)	0.0494 (14)	0.0470 (13)	−0.0051 (12)	0.0068 (11)	−0.0079 (11)
C18	0.0809 (19)	0.0515 (14)	0.0466 (14)	−0.0031 (13)	0.0096 (13)	−0.0091 (11)
C19	0.083 (2)	0.0556 (15)	0.0487 (14)	−0.0081 (14)	0.0050 (13)	−0.0121 (12)
C20	0.098 (2)	0.0628 (17)	0.0465 (14)	−0.0181 (15)	0.0105 (14)	−0.0130 (12)
C21	0.084 (2)	0.0624 (16)	0.0420 (13)	−0.0190 (14)	0.0143 (13)	−0.0109 (12)
O5	0.125 (4)	0.120 (4)	0.123 (4)	0.0016 (19)	0.0008 (19)	0.0101 (19)
C22	0.106 (3)	0.105 (3)	0.107 (3)	0.0010 (10)	0.0003 (10)	−0.0001 (10)
C23	0.107 (3)	0.104 (3)	0.106 (3)	0.0061 (19)	0.0028 (19)	0.0021 (19)
O5A	0.070 (2)	0.060 (2)	0.067 (2)	−0.0011 (15)	0.0075 (16)	0.0087 (16)
C22A	0.098 (3)	0.096 (3)	0.099 (3)	0.0021 (10)	0.0001 (10)	0.0004 (10)
C23A	0.098 (4)	0.094 (3)	0.100 (3)	0.0024 (19)	−0.0061 (19)	0.0016 (19)

### 5-[(3-{2-[Bis(propan-2-yl)azaniumyl]ethyl}-1H-indol-4-yl)oxy]-5-oxopentanoate ethanol monosolvate (II) Geometric parameters (Å, º)

**Table:**

O1—C6	1.409 (3)	C13—H13B	0.9600
O1—C17	1.356 (3)	C13—H13C	0.9600
O2—C17	1.183 (3)	C14—H14	0.9800
O3—C21	1.234 (3)	C14—C15	1.520 (4)
O4—C21	1.264 (3)	C14—C16	1.510 (4)
N1—C1	1.360 (4)	C15—H15A	0.9600
N1—C2	1.359 (3)	C15—H15B	0.9600
N1—H1	0.853 (10)	C15—H15C	0.9600
N2—C10	1.505 (3)	C16—H16A	0.9600
N2—C11	1.521 (4)	C16—H16B	0.9600
N2—C14	1.517 (3)	C16—H16C	0.9600
N2—H2	0.90 (3)	C17—C18	1.493 (3)
C1—H1A	0.9300	C18—H18A	0.9700
C1—C8	1.359 (3)	C18—H18B	0.9700
C2—C3	1.386 (4)	C18—C19	1.509 (3)
C2—C7	1.420 (3)	C19—H19A	0.9700
C3—H3	0.9300	C19—H19B	0.9700
C3—C4	1.370 (4)	C19—C20	1.498 (3)
C4—H4	0.9300	C20—H20A	0.9700
C4—C5	1.400 (4)	C20—H20B	0.9700
C5—H5	0.9300	C20—C21	1.518 (4)
C5—C6	1.358 (4)	O5—H5A	0.8200
C6—C7	1.397 (3)	O5—C22	1.439 (8)
C7—C8	1.427 (4)	C22—H22A	0.9700
C8—C9	1.499 (3)	C22—H22B	0.9700
C9—H9A	0.9700	C22—C23	1.496 (8)
C9—H9B	0.9700	C23—H23A	0.9600
C9—C10	1.521 (3)	C23—H23B	0.9600
C10—H10A	0.9700	C23—H23C	0.9600
C10—H10B	0.9700	O5A—H5AA	0.8200
C11—H11	0.9800	O5A—C22A	1.420 (8)
C11—C12	1.517 (4)	C22A—H22C	0.9700
C11—C13	1.514 (5)	C22A—H22D	0.9700
C12—H12A	0.9600	C22A—C23A	1.470 (8)
C12—H12B	0.9600	C23A—H23D	0.9600
C12—H12C	0.9600	C23A—H23E	0.9600
C13—H13A	0.9600	C23A—H23F	0.9600
			
C17—O1—C6	116.79 (18)	C15—C14—H14	107.3
C1—N1—H1	124 (2)	C16—C14—N2	110.5 (2)
C2—N1—C1	109.1 (2)	C16—C14—H14	107.3
C2—N1—H1	127 (2)	C16—C14—C15	112.3 (3)
C10—N2—C11	111.28 (19)	C14—C15—H15A	109.5
C10—N2—C14	112.0 (2)	C14—C15—H15B	109.5
C10—N2—H2	106.1 (17)	C14—C15—H15C	109.5
C11—N2—H2	107.4 (18)	H15A—C15—H15B	109.5
C14—N2—C11	113.6 (2)	H15A—C15—H15C	109.5
C14—N2—H2	106.0 (17)	H15B—C15—H15C	109.5
N1—C1—H1A	124.5	C14—C16—H16A	109.5
C8—C1—N1	111.1 (3)	C14—C16—H16B	109.5
C8—C1—H1A	124.5	C14—C16—H16C	109.5
N1—C2—C3	131.0 (2)	H16A—C16—H16B	109.5
N1—C2—C7	106.9 (2)	H16A—C16—H16C	109.5
C3—C2—C7	122.1 (2)	H16B—C16—H16C	109.5
C2—C3—H3	120.9	O1—C17—C18	111.0 (2)
C4—C3—C2	118.1 (2)	O2—C17—O1	122.7 (2)
C4—C3—H3	120.9	O2—C17—C18	126.3 (2)
C3—C4—H4	119.3	C17—C18—H18A	108.9
C3—C4—C5	121.3 (3)	C17—C18—H18B	108.9
C5—C4—H4	119.3	C17—C18—C19	113.5 (2)
C4—C5—H5	120.0	H18A—C18—H18B	107.7
C6—C5—C4	120.0 (3)	C19—C18—H18A	108.9
C6—C5—H5	120.0	C19—C18—H18B	108.9
C5—C6—O1	120.4 (2)	C18—C19—H19A	109.0
C5—C6—C7	121.4 (2)	C18—C19—H19B	109.0
C7—C6—O1	118.2 (2)	H19A—C19—H19B	107.8
C2—C7—C8	107.4 (2)	C20—C19—C18	113.1 (2)
C6—C7—C2	117.0 (2)	C20—C19—H19A	109.0
C6—C7—C8	135.6 (2)	C20—C19—H19B	109.0
C1—C8—C7	105.6 (2)	C19—C20—H20A	108.4
C1—C8—C9	124.9 (2)	C19—C20—H20B	108.4
C7—C8—C9	128.8 (2)	C19—C20—C21	115.7 (2)
C8—C9—H9A	110.0	H20A—C20—H20B	107.4
C8—C9—H9B	110.0	C21—C20—H20A	108.4
C8—C9—C10	108.65 (19)	C21—C20—H20B	108.4
H9A—C9—H9B	108.3	O3—C21—O4	122.7 (2)
C10—C9—H9A	110.0	O3—C21—C20	121.0 (2)
C10—C9—H9B	110.0	O4—C21—C20	116.3 (2)
N2—C10—C9	114.35 (19)	C22—O5—H5A	109.5
N2—C10—H10A	108.7	O5—C22—H22A	110.0
N2—C10—H10B	108.7	O5—C22—H22B	110.0
C9—C10—H10A	108.7	O5—C22—C23	108.4 (8)
C9—C10—H10B	108.7	H22A—C22—H22B	108.4
H10A—C10—H10B	107.6	C23—C22—H22A	110.0
N2—C11—H11	108.1	C23—C22—H22B	110.0
C12—C11—N2	111.6 (2)	C22—C23—H23A	109.5
C12—C11—H11	108.1	C22—C23—H23B	109.5
C13—C11—N2	110.0 (2)	C22—C23—H23C	109.5
C13—C11—H11	108.1	H23A—C23—H23B	109.5
C13—C11—C12	111.0 (3)	H23A—C23—H23C	109.5
C11—C12—H12A	109.5	H23B—C23—H23C	109.5
C11—C12—H12B	109.5	C22A—O5A—H5AA	109.5
C11—C12—H12C	109.5	O5A—C22A—H22C	111.4
H12A—C12—H12B	109.5	O5A—C22A—H22D	111.4
H12A—C12—H12C	109.5	O5A—C22A—C23A	101.9 (7)
H12B—C12—H12C	109.5	H22C—C22A—H22D	109.3
C11—C13—H13A	109.5	C23A—C22A—H22C	111.4
C11—C13—H13B	109.5	C23A—C22A—H22D	111.4
C11—C13—H13C	109.5	C22A—C23A—H23D	109.5
H13A—C13—H13B	109.5	C22A—C23A—H23E	109.5
H13A—C13—H13C	109.5	C22A—C23A—H23F	109.5
H13B—C13—H13C	109.5	H23D—C23A—H23E	109.5
N2—C14—H14	107.3	H23D—C23A—H23F	109.5
N2—C14—C15	111.9 (2)	H23E—C23A—H23F	109.5
			
O1—C6—C7—C2	179.04 (19)	C6—O1—C17—O2	0.6 (4)
O1—C6—C7—C8	0.8 (4)	C6—O1—C17—C18	−178.4 (2)
O1—C17—C18—C19	−176.7 (2)	C6—C7—C8—C1	179.0 (3)
O2—C17—C18—C19	4.3 (5)	C6—C7—C8—C9	8.9 (4)
N1—C1—C8—C7	0.0 (3)	C7—C2—C3—C4	−0.1 (4)
N1—C1—C8—C9	170.6 (2)	C7—C8—C9—C10	86.1 (3)
N1—C2—C3—C4	178.3 (3)	C8—C9—C10—N2	180.0 (2)
N1—C2—C7—C6	−179.8 (2)	C10—N2—C11—C12	−178.7 (3)
N1—C2—C7—C8	−1.1 (3)	C10—N2—C11—C13	57.7 (3)
C1—N1—C2—C3	−177.5 (3)	C10—N2—C14—C15	62.8 (3)
C1—N1—C2—C7	1.1 (3)	C10—N2—C14—C16	−63.1 (3)
C1—C8—C9—C10	−82.3 (3)	C11—N2—C10—C9	−105.4 (3)
C2—N1—C1—C8	−0.7 (3)	C11—N2—C14—C15	−64.3 (3)
C2—C3—C4—C5	1.0 (4)	C11—N2—C14—C16	169.8 (2)
C2—C7—C8—C1	0.7 (3)	C14—N2—C10—C9	126.3 (2)
C2—C7—C8—C9	−169.4 (2)	C14—N2—C11—C12	−51.2 (3)
C3—C2—C7—C6	−1.0 (3)	C14—N2—C11—C13	−174.8 (2)
C3—C2—C7—C8	177.7 (2)	C17—O1—C6—C5	−87.0 (3)
C3—C4—C5—C6	−0.8 (4)	C17—O1—C6—C7	95.2 (3)
C4—C5—C6—O1	−178.1 (2)	C17—C18—C19—C20	176.7 (3)
C4—C5—C6—C7	−0.4 (4)	C18—C19—C20—C21	172.6 (3)
C5—C6—C7—C2	1.2 (3)	C19—C20—C21—O3	−5.6 (5)
C5—C6—C7—C8	−177.0 (3)	C19—C20—C21—O4	175.3 (3)

### 5-[(3-{2-[Bis(propan-2-yl)azaniumyl]ethyl}-1H-indol-4-yl)oxy]-5-oxopentanoate ethanol monosolvate (II) Hydrogen-bond geometry (Å, º)

**Table:**

*D*—H···*A*	*D*—H	H···*A*	*D*···*A*	*D*—H···*A*
N2—H2···O4^i^	0.90 (3)	1.79 (3)	2.686 (3)	177 (3)
N1—H1···O3^ii^	0.85 (1)	1.91 (1)	2.751 (3)	167 (3)
O5—H5*A*···O4^iii^	0.82	1.97	2.692 (10)	147
O5*A*—H5*AA*···O4^iii^	0.82	1.95	2.732 (6)	160

Symmetry codes: (i) −*x*+1, −*y*+1, −*z*+1; (ii) −*x*+1, *y*−1/2, −*z*+1/2; (iii) −*x*, −*y*+1, −*z*+1.
